# Assessment of Financial Toxicity Among Patients With Advanced Lung Cancer in Western China

**DOI:** 10.3389/fpubh.2021.754199

**Published:** 2022-01-12

**Authors:** Tianqi Xu, Leidi Xu, Hangtian Xi, Yong Zhang, Ying Zhou, Ning Chang, Wenhui Yang, Yan Zhang, Ming Wang, Qing Ju, Xuemin Yang, Xiangxiang Chen, Yinggang Che, Fulin Chen, Shuoyao Qu, Jian Zhang

**Affiliations:** ^1^Department of Pulmonary Medicine, Xijing Hospital, Air Force Medical University, Xi'an, China; ^2^School of Public Health, Air Force Medical University, Xi'an, China; ^3^Szechwan Maternal and Child Care Service Centre, Szechwan, China; ^4^College of Life Sciences, Northwest University, Xi'an, China

**Keywords:** financial burden, financial toxicity, medical cost, health-related quality of life (HRQL), lung cancer

## Abstract

**Background:** Lung cancer is the leading source of cancer-caused disability-adjusted life years. Medical cost burden impacts the well-being of patients through reducing income, cutting daily expenses, curtailing leisure activities, and depleting exhausting savings. The COmprehensive Score for Financial Toxicity (COST) was created and validated by De Souza and colleagues. Our study intends to measure the financial burdens of cancer therapy and investigate the link between financial toxicity and health-related quality of life (HRQoL) in an advanced lung cancer population.

**Methods:** Patients aged ≥ 18 years with confirmed stage III to IV lung cancer were eligible. The COST questionnaire verified by de Souza et al. was used to identify financial toxicity. Multivariable linear regression analysis with log transformation univariate analysis and Pearson correlations were used to perform the analysis.

**Results:** The majority of the patients (90.8%, *n* = 138/152) had an annual income of $50,000 ($7,775). The cohort's insurance situation was as follows: 64.5% of the cohort had social insurance, 20.4% had commercial insurance, and 22.0% had both. Patients who were younger age (50–59, *P* < 0.001), employed but on sick leave, and had lower income reported increased levels of financial toxicity (*P* < 0.05). The risk factors for high financial toxicity: (i) younger age (50–59), (ii) <1 month of savings, and (iii) being employed but on sick leave. Increased financial toxicity is moderately correlated with a decrease in QoL.

**Conclusion:** Poorer psychological status and specific demographics are linked to increased financial toxicity (lower COST). Financial toxicity has a modest relationship with HRQoL and may have a clear link with HRQoL measurements.

## Introduction

In most nations, lung cancer is the primary reason for cancer-caused disability-adjusted life years (DALYs) (1, 2). Patients with lung cancer face considerable medical costs and service utilization (3–5). The financial burden on patients with lung cancer includes both direct costs and indirect expenditures, such as transportation and lost income (out of work or on sick leave) (3, 6). Medical cost burden has an influence on the well-being of patients through reducing income, cutting daily spending, leisure activities, and eliminating savings. Financial hardship has been identified as an independent risk factor for mortality since it might lead to bankruptcy (7).

The phrase financial toxicity was coined in order to draw attention to financial stress and its detrimental implications. It refers to a side effect of cancer treatment that is comparable to nausea and alopecia (8). The COmprehensive Score for Financial Toxicity (COST) was then developed and validated by De Souza et al. (9, 10). The COST questionnaire has 11 elements with values ranging from 0 to 44. They validated it using a population from North America. Lower COST ratings in De Souza's study indicated a higher degree of financial toxicity. Then, studies on financial toxicity revealed that increased financial toxicity was connected with a lower Health-Related Quality of Life (HRQoL) and increased psychological distress. Meanwhile, it has an effect on cancer therapy. To cut expenses, patients take less medication than advised, use over-the-counter medications, and take medicine prescribed by others (11).

China covers approximately 20% of the world's population. As a developing country, China is confronted with the most challengeable medical funding issues. In fact, China has knitted the global biggest network of universal basic medical insurance and established a healthcare service system that encompasses both urban and rural areas. Social medical insurance in China contains three types which are the basic medical insurance for urban employees, basic medical insurance for urban residents, and the new rural cooperative medical system. By the end of 2016, China's social medical insurance has reached over 1.3 billion people across the country, accounting for more than 95% of the total population. Nonetheless, updating the policy level of medical insurance is still required.

Policy reforms in the Chinese medical care insurance system should maximize individual advantages. However, before it changes, access to a better understanding of the current state of financial toxicity as well as its negative impact on the QoL of patients must be opened, which could improve the interventions aimed at reducing financial distress, thus improving quality care and policy optimization. Our study intends to examine the economic costs of cancer care in the advanced lung cancer population using a validated financial toxicity tool (COST questionnaire). We also investigated the link between financial toxicity and HRQoL. This work might aid in improving resource allocation for early intervention by inferring the risk variables of financial damage in patients with lung cancer (12).

## Cohort and Methods

### Cohort

Patients aged ≥ 18 years with stage III to IV(AJCC, 8th edition) lung cancer (non-small and small cell lung cancer) who require continued multimodality therapies throughout time were eligible. The following criteria were included: (1) stage III–IV lung cancer without the opportunity for surgery, (2) ongoing anti-tumor therapies of drugs and injections, and (3) agree to receive this interview. The excluding criteria contained: (1) stage I–II lung cancer with the opportunity for surgery, (2) malignancies of metastasis to the lung, (3) participating in other clinical trials, and (4) refuse to take the interview. We excluded the stage II group of patients with lung cancer who missed the opportunity to receive thoracic surgery due to few of them (3 cases) appeared in our study. Ethics approval was acquired from the Human Research Ethics Committee of Xijing Hospital (KY20202077-C-1).

### Variables and Outcomes

Information of the performance, tumor stage, and histological diagnose of the patients were extracted from electronic medical records from the Xijing database. We obtained direct information from patients on their marital status, job, family income, and household savings. The baseline financial situation of the patients was determined using previously published financial toxicity (FT) questionnaires (Supplementary Table 1) and translated into Mandarin (13). FT was evaluated by the COST instrument (10), translated into Mandarin. Financial toxicity was rated as strong by a score larger than the cohort's median COST (14). The Functional Assessment of Cancer Therapy-Lung (FACT-L) questionnaire was used to measure the HRQoL (15, 16). The FACT-L instrument contains two parts, which are a common module for all patients with cancer (a 27 item cancer therapy function evaluation general scale, FACT-G) and a lung cancer-specific module (a 9-item lung cancer additional concern part), ranging from 0 to 136. It has been determined that a higher FACT score indicates a higher QoL.

### Statistical Analysis

We summarized the baseline and financial status by descriptive statistics. To compare the average COST scores in patients, we used multivariable linear regression analysis with log transformation. We identified significant covariates in multivariable regression analysis (*P* ≤ 0.10 was considered clinical significance). The Pearson correlation was used to assess the correlations between COST and FACT scores. If the coefficient was 0.20 to 0.39, the correlation was regarded as mild, 0.4–0.59 moderate, 0.60–0.79 strong, and ≥ 0.80 very strong. *P* < 0.05 (two-tailed) was considered statistically significant. All statistical analyses were performed using SPSS 22.0 (IBM, New York, USA).

## Results

**Figure 1:**
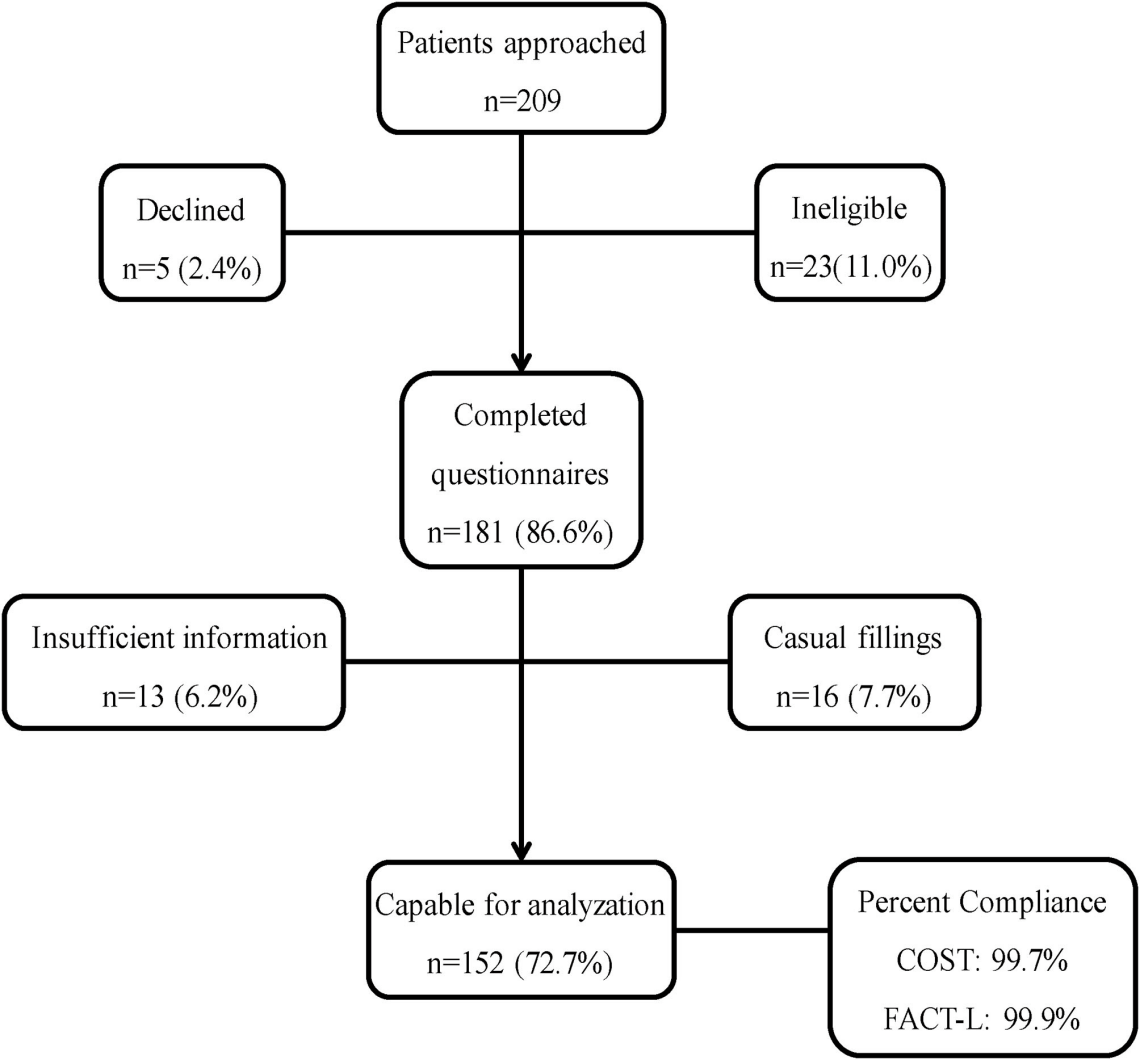
Patient Recruitment Process. COST, the COmprehensive Score for Financial Toxicity; FACT-L, the Functional Assessment of Cancer Therapy-Lung questionnaire.

We accessed the cohort from September 2019 to January 2021. Figure 1 showed the recruitment process. The baseline of the cohort included in the analysis is summarized in Table 1. We recruited 209 potential participants, of which 152 were eligible. The median age was 62.1 years (range 32–84 years). A total of 65.1% (*n* = 99) had metastasis and 44.9% (*n* = 53) had localized disease. The majority of patients had non-small cell carcinoma histology (63.2%, *n* = 96/152) followed by small cell carcinoma histology (27.0%, *n* = 41/152) and other lung cancer histology (9.8%, *n* = 15/152). The median value of the COST scores was 25.5 (range 4–42, mean ± SD 9.7 ± 0.8).

**Table 1:** Patient demographics.

**Characteristics**	**Values (*n*[%])**
Age (y)	
Median	62.1
Range	32–84
Sex	
Male	81 (53.3)
Female	71 (46.7)
Marital state	
Married	144 (94.7)
Unmarried	8 (5.3)
Current smoker	
Yes	59 (38.8)
No	93 (61.2)
Insurance type	
Social insurance	98 (64.5)
Commercial insurance	31 (20.4)
Social & Commercial insurance	22 (14.5)
No insurance	1 (0.1)
Primary place of residence	
Owner/occupier—no mortgage	95 (62.6)
Owner/occupier with mortgage	45 (29.6)
Renting (>3 years)	8 (5.2)
Living with family/friends (>10 years)	2 (1.3)
Other	2 (1.3)
Current employment status	
Working full time	15 (9.9)
Working part time	8 (5.2)
Retired	101 (66.4)
Unemployed	27 (17.8)
Other (student, homemaker)	1 (0.7)
Change in employment status	
Yes	132 (86.8)
No	20 (13.2)
Household income per year (CNY)	
<20,000	36 (23.7)
20,000–49,999	102 (67.1)
50,000–99,999	9 (5.9)
>100,000	5 (3.3)
Current household savings (CNY)	
<1 month (reference)	63 (41.4)
1–6 months	41 (27.0)
7–12 months	16 (10.5)
>year	32 (21.1)
Stage	
III	66 (43.4)
IV	61 (40.1)
Histology	
Small-cell carcinoma	41 (27.0)
Non-small cell carcinoma	96 (63.2)
Other	15 (9.8)

*CNY, China Yuan*.

### Financial State

Of the financial perspectives, most of the patients (90.8%, *n* = 138/152) had an household income < ¥50,000 ($7,775) per year. The insurance condition of the cohort was that the majority of the cohort had social insurance (64.5%, *n* = 98/152), 20.4% of them had commercial insurance (*n* = 31/152), 22.0% of them had both (*n* = 22/152). Only one patient *(0.1*%) had no insurance. Housing mortgage holds the overwhelming majority of the loans of Chinese families in modern China, in all probability determining the disposable income of a family. Thus, we brought residence condition to reflect the financial state of the patient as its close relation to individual financial pressure. Due to elderly residences, most of the patients were the owner of the house without a mortgage (62.6%, *n* = 95/152). While the occupiers with a mortgage were 29.6% (*n* = 45/152). A total of 14.5% (*n* = 22/152) of the patients had retirement salaries to meet medical costs. Most of our cohort was retired (64.1%, *n* = 84/152). Of others, 59.6% (*n* = 28/152) changed in income when the cancer treatment started.

### Variables Associated With FT

Univariate analysis of variables related to FT was described in Table 2. Patients at a younger age (50–59, *P* < 0.001), employed but on sick leave, and had a lower income had increased levels of FT (*P* < 0.05). In multivariable modeling, we adjusted for potentially confounding variables and discovered patients who had <1 months' worth of household savings turned to have higher financial toxicity (*P* < 0.05).

**Table 2:** Patient characteristics and COST outcomes.

**Characteristics**	**Univariate analysis**		**Multivariable analysis**	
	**Coefficient (95% CI)**	***P*-value**	**Coefficient (95% CI)**	***P*-value**
Age (y)				
<50 (reference)				
50–59	10.1 (5.7 to 19.7)	<0.001	−5.6 (−9.8 to 8.6)	0.82
60–69	5.6 (4.2 to 8.9)	0.02	0.2 (−7.0 to 7.3)	0.56
70–79	1.3 (−2.2 to 5.1)	0.09	2.7 (−5.8 to 7.9)	0.33
>80	2.8 (−4.5 to 6.2)	0.16	1.2 (−8.9 to 10.3)	0.29
Sex				
Female (reference)				
Male	0.54 (−2.6 to 3.1)	0.49	2.7 (−0.9 to 4.3)	0.22
Marital state				
Married (reference)				
Unmarried	−5.7 (−6.4 to 2.8)	0.06	−3.3 (−4.1 to 2.2)	0.34
ECOG performance status				
0 (reference)				
1	1.0 (−5.3 to 6.6)	0.66	0.23 (−3.2 to 4.1)	0.82
≥2	−1.7 (−5.5 to 4.2)	0.46	−0.4 (−4.5 to 4.7)	0.76
Insurance type				
Social Insurance (reference)				
Commercial insurance	2.1 (−3.3 to 5.9)	0.52	−2.3 (−7.6 to 5.7)	0.43
Social and Commercial insurance	−8.6 (−13.8 to 4.5)	0.003	−4.6 (−6.3 to 0.2)	0.02
Current employment status				
Working full time/part time (reference)				
Employed, on sick leave	3.5 (−3.2 to 6.9)	0.05	5.6 (−6.8 to 9.9)	0.59
Retired	−6.2 (0.7 to 18.4)	0.04	1.6 (−6.8 to 9.9)	0.63
Unemployed	8.1 (−0.8 to 9.3)	0.08	1.2 (−7.8 to 10.2)	0.86
Household income per year (CNY)				
<20,000 (reference)				
20,000–49,999	2.8 (−6.2 to 8.9)	0.33	−2.8 (−4.6 to 7.0)	0.78
50,000–99,999	6.2 (−1.3 to 7.9)	0.55	−1.3 (−2.2 to 3.4)	0.59
>100,000	9.7 (3.8 to 13.4)	0.01	3.4 (−3.2 to 6.1)	0.42
Household savings				
<1 month (reference)				
1–6 months	1.9 (−1.0 to 2.8)	0.18	6.2 (0.3 to 8.6)	0.06
7–12 months	8.5 (2.8 to 14.1)	<0.001	4.5 (−1.2 to 6.2)	0.34
>1 year	11.3 (13 to 22.1)	<0.001	8.1 (6.4 to 15.4)	<0.001

*ECOG, eastern cooperative oncology group; CNY, China Yuan*.

When compared with this value, patients who had >1–6, 7–12 months' and >1 years' worth of savings scored 3.9 points (95% CI: 2.1 to 4.9, *P* = 0.06), 10.4 points (95% CI: 5.0 to 16.1, *P* < 0.05), and 22.6 points (95% CI: 11.6 to 26.3, *P* < 0.001) higher COST scores, respectively. As to employment condition, patients on sick leave had increased financial toxicity compared with employed patients (8.3, 95% CI: 6.3 to 11.6, *P* = 0.04) or retired (7.2, 95% CI: 4.2 to 14.6, *P* = 0.02).

In Table 2, we identified risk factors for higher FT scores: (i) younger age (50–59), (ii) being employed but on sick leave, and (iii) with <1 month of savings. A total of 54.9% (*n* = 52) of our cohort had no risk factors. In total, 32.8% (*n* = 61) had one risk factor and 8.4% (*n* = 24) had two risk factors. Lastly, 3.8% (*n* = 15) had all three risk factors.

### Association Between FT and QoL

**Figure 2:**
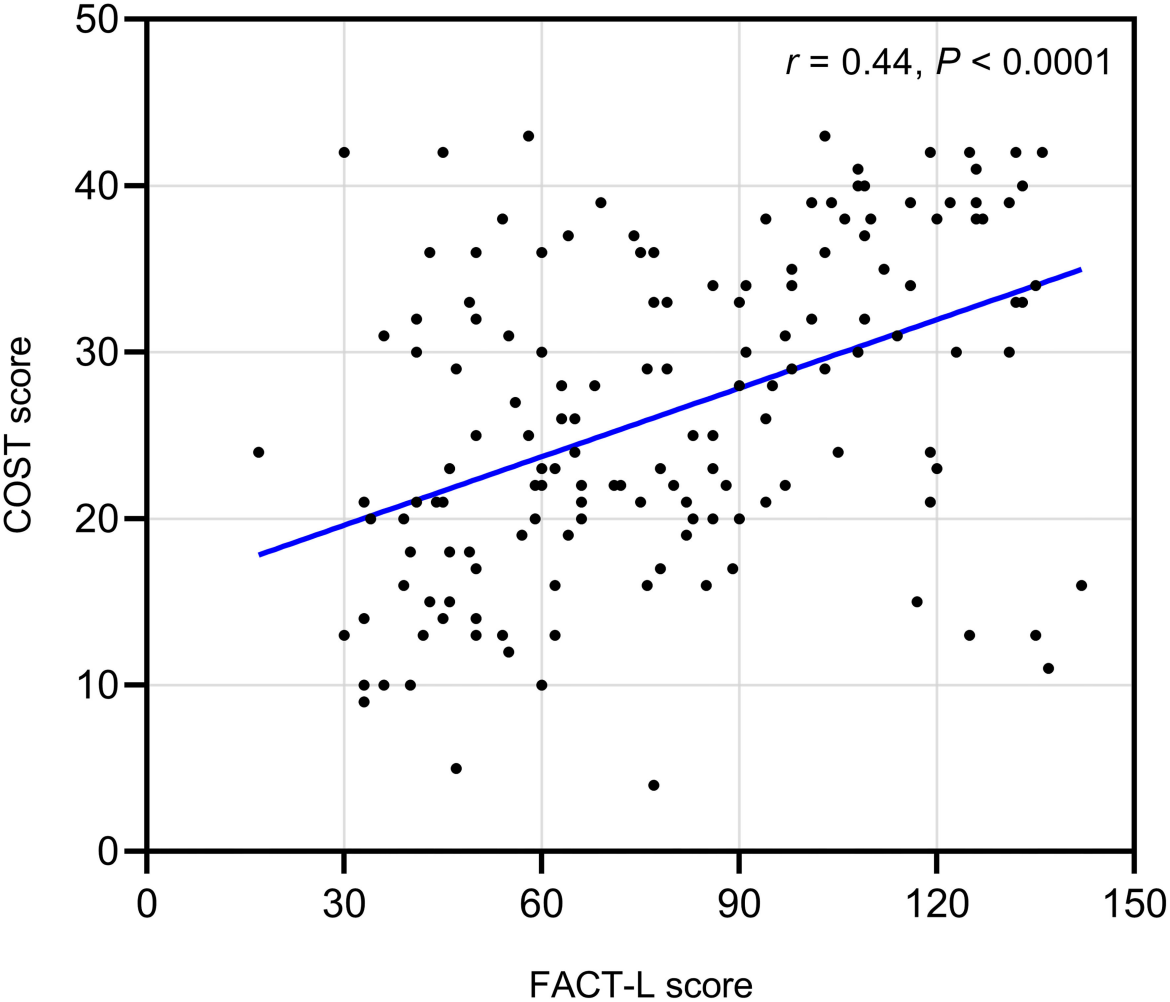
Correlation between financial toxicity and health-related quality of life. Increased financial toxicity (lower COST) is moderately associated with decreased quality of life (lower FACT-L). Pearson correlation coefficient*:0.4*4, *P* < 0.0001.

The r-value of the COST score and FACT-L was 0.44 (*P* < 0.0001), which inferred a moderate correlation of the two variables (Figure 2), indicating that increased FT (lower COST) is moderately associated with decreased QoL (lower FACT-L).

## Discussion

Most of the patients with cancer received their treatment in China's public hospitals, while private ones contributed less in treating malignancies. Similar to previous studies (10, 17), this study recruited patients from a public hospital in West China who had all types of health insurance (social insurance, commercial insurance, and both). In contrast to Australia‘s and certain European nations' healthcare systems, China lacks comprehensive healthcare insurance coverage. Nonetheless, with 95% of its population covered, China's healthcare system may not be overly reliant on commercial insurance, in contrast to the United States, where around 33% of its population is uninsured (18, 19).

The cost of anti-tumor medications has decreased as a result of China's new policy on imported pharmaceuticals and the introduction of domestically made ones. However, ancillary expenditures for cancer therapy, such as transportation and lodging, vary by city. As a result, assessing the absolute quantities of the medical expenses of the patient directly may not be enough to assess the financial strain on the family of the patient in China. We examined FT in a sample of Chinese patients with lung cancer using COST established by De Souza et al. Our analysis discovered that patient demographic characteristics related to greater FT included younger age, being employed but on sick leave, and having smaller funds. These findings may aid in identifying potential patients who are at a higher risk of experiencing negative health outcomes.

Previous research has shown that anti-tumor medications cost higher for patients with advanced cancer (20), and they displayed a more severe financial toxicity than physical, familial, and emotional distress (21–23). Furthermore, our findings corroborated the idea that FT had a modest connection with HRQoL but did not precisely assess it, suggesting that FT may have a detrimental influence on patient well-being (24). Our findings indicated that the 50- to 59-year-old population was the most vulnerable to financial toxicity, which might be attributed to their general economic situation in modern China. Chinese residents aged 50–59 may face the greatest financial strain in society. It is commonly understood in Chinese culture that one of the goals of wealth is to provide care for the old. Chinese residents aged 50–59 typically bear this obligation since their parents aged 80–90 are incapacitated for life. They should share their time and wealth with their parents. Meanwhile, because their children aged 20–30 whose salary could not cover their mortgage payment, residents aged 50–59 must assist their children on this issue. In such cases, if they were diagnosed with lung cancer and subsequently saddled with the related medical bills, their life may be made more difficult.

As mentioned, the Chinese healthcare system is mostly based on social insurance, which shows the Chinese government's constant efforts. However, even with commercial health insurance, China's healthcare system only covers hospital-related medical costs (drugs and inpatient stays), not transportation or lodging for specialist consultations. Meanwhile, according to the health care policy, residents in China must pay for medical bills in advance and then wait for reimbursement from the health insurance agency, which may make household savings critical for Chinese families. Remote inhabitants, on the other hand, may have a greater financial burden as a result of increased commuting costs and lengthier sick absences. Regrettably, our study was unable to substantiate the higher FT among patients residing in distant places.

In western countries, patients with cancer are able to get access to cancer care teams, including an oncologist, nurse practitioner, and case manager/ financial counselor. When they have any problems in receiving cancer therapies, they can contact one or some in the team to get professional assistance. While in China, such a team merely contains oncologists and nurse practitioners, which may cause bewilderment in facing financial issues associated with medical care, the oncologist and nurse practitioner are unqualified in answering the financial questions of patients. Oncologists in China feel the costs of medical care are important, yet they are poorly prepared to discuss costs with patients in the clinic. The customer representatives of large pharmaceutical companies such as Roche, Pfizer, and Merck may play this role, yet inevitably mixed with the company's interests when answering financial questions. Hence, there is an urgent need for the emergence of roles such as “financial counselor/case manager” in the cancer care team of China, who should be multi-professional such as medicine and economics, qualified to offer professional help in giving or assessing financial issues during cancer therapies.

The medical data in China is hospital-independent. Unfortunately, there is no integrated platform for researches, making nationwide research on the financial burden upon patients with cancer very difficult. One weakness of our study was that it remained single-centered. A multi-centered cohort may reach a more realistic conclusion. Furthermore, as previously stated, our study did not include measuring FT in patients residing in distant locations, which may result in worse cancer outcomes.

This study aimed to raise awareness of the financial burden on lung cancer patients among policymakers and doctors, as well as to better identify individuals at the highest risks for cancer-related financial toxicity. It is undeniable that the entire society should work together to relieve such a burden. In order to address the lack of a countrywide platform holding medical data and to enhance policy optimization, future research should encompass additional locations of China as well as more forms of cancer.

## Conclusion

The financial burden of cancer and cancer treatment has become more visible, as does the way to analyze it. Verified studies guarantee that accurate conclusions are provided in such fields. This study revealed that FACT COST is a relevant and reliable tool for lung cancer patients in China. Poorer psychological status and specific demographics are associated with increased financial toxicity (lower COST). Financial toxicity is modestly connected to HRQoL and may have a discernible relationship with HRQoL assessments.

## Data Availability Statement

The raw data supporting the conclusions of this article will be made available by the authors, without undue reservation.

## Ethics Statement

The studies involving human participants were reviewed and approved by Human Research Ethics Committee of Xijing Hospital, Approve Number: KY20202077-C-1. The patients/participants provided their written informed consent to participate in this study.

## Author Contributions

JZ contributed to the conception of the study and performed constructive discussions. TX, YiZ, and YoZ performed the data collection and analysis. TX and LX wrote the manuscript. WY, HX, YC, and YaZ contributed to data analysis and manuscript preparation. SQ, FC, NC, MW, QJ, XY, and XC contributed to the revised manuscript. All authors contributed to the article and approved the submitted version.

## Conflict of Interest

The authors declare that the research was conducted in the absence of any commercial or financial relationships that could be construed as a potential conflict of interest.

## Publisher's Note

All claims expressed in this article are solely those of the authors and do not necessarily represent those of their affiliated organizations, or those of the publisher, the editors and the reviewers. Any product that may be evaluated in this article, or claim that may be made by its manufacturer, is not guaranteed or endorsed by the publisher.
