# Supplementary material for: Assessment of Financial Toxicity Among Patients With Advanced Lung Cancer in Western China
Source: Front Public Health. 2022 Jan 12;9:754199. doi: 10.3389/fpubh.2021.754199 (PMC8790143; doi:10.3389/fpubh.2021.754199)
Supplement: Supplementary file 1 [file Data_Sheet_1.docx]

**Supplementary Table 1**. Financial State and Health Insurance Questionnaire

1. **What is your current work status?**

Please choose **only one** of the following:

| ○ | Full-time Job | | |
| --- | --- | --- | --- |
| ○ | Part-time Job | | |
| ○ | Self employed | | |
| ○ | Retired | | |
| ○ | Employed, on sick leave due to lung cancer ( > 1 week) | | |
| ○ | Unemployed | | |
| ○ | Other: |  |  |

1. **If EMPLOYED, what is your occupation (including pre-retirement)?**

(For example: office work/farming/business/engineering, etc.)

Please write your answer here:

|  |
| --- |

1. **If EMPLOYED, how many HOURS per week do you usually work?**

Please choose **only one** of the following::

| ○ | < 40 hours |
| --- | --- |
| ○ | 40-60 hours |
| ○ | > 60 hours |

1. **What is your current living situation?**

Please choose **the closest to your condition** of the following:

| ○ | Owner/occupier - no mortgage | | |
| --- | --- | --- | --- |
| ○ | Owner/occupier with mortgage | | |
| ○ | Renting ( > 3 years) | | |
| ○ | Living with family/friends | | |
| ○ | Other: |  |  |

1. **What is your current household SAVINGS (CNY)?**

Please choose **the closest to your condition** of the following:

| ○ | < 1 month |
| --- | --- |
| ○ | 1 ~ 6 months |
| ○ | 7 ~ 12 months |
| ○ | > 1 year |

1. **What is your current** **household INCOME per year (CNY)?**

Please choose **the closest to your condition** of the following:

| ○ | < 20,000 |
| --- | --- |
| ○ | 20,000 ~ 49,999 |
| ○ | 50,000 ~ 99,999 |
| ○ | > 100,000 |

1. **What type is your current** **medical INSURANCE?**

Please choose **the closest to your condition** of the following:

| ○ | Social medical insurance |
| --- | --- |
| ○ | Commercial medical insurance |
| ○ | Both |
| ○ | None |

1. **How does your current** **medical insurance cover your medical cost?**

Please choose **the closest to your condition** of the following:

| ○ | ≤ 40% |
| --- | --- |
| ○ | 40%~69% |
| ○ | 70%~89% |
| ○ | ＞90% |

1. **Now please think of your work experiences over the past 4 weeks (28 days). Have any of the following state meet your condition?**

Please choose **the closest to your condition** of the following:

| ○ | I cannot work normally due to lung cancer < 1 day |
| --- | --- |
| ○ | I cannot work normally due to other reasons < 1 day |
| ○ | I cannot work normally due to lung cancer ≥ 1 day |
| ○ | I cannot work normally due to other reasons ≥ 1 day |
| ○ | None of those happened |

1. **Have you experienced any of the following changes at work since your cancer diagnosis?**

Please choose **the closest to your condition** of the following:

| ○ | My current situation has not changed since my diagnosis | | |
| --- | --- | --- | --- |
| ○ | My work hours decreased | | |
| ○ | I ceased working / retired | | |
| ○ | Other: |  |  |

1. **One a scale from 0 to 10 where 0 is the worst job performance anyone could have at your job and 10 is the performance of a top worker, how would you rate your usual performance to most workers in a job similar to yours?**


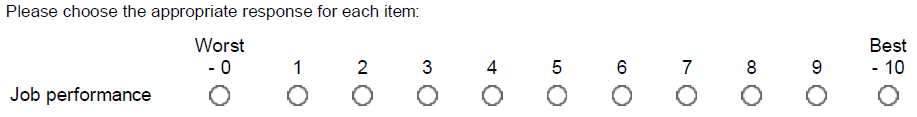


1. **How would you rate your working performance of recent 4 weeks?**


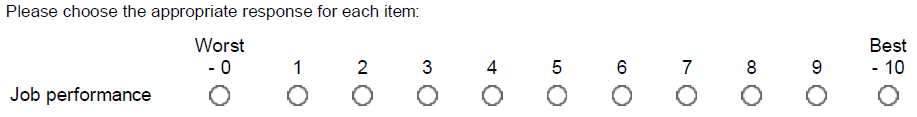


1. **Have you experienced coming in early, going home late, or working on your day off since your cancer diagnosis?**

Please choose **the closest to your condition** of the following:

| ○ | Yes |
| --- | --- |
| ○ | No |

This is the end of the survey.

Thank you again for taking the time to complete it and please accept our best wishes for your health.

Thank you for completing this survey.
